# Non-small cell lung cancer (NSCLC): A review of risk factors, diagnosis, and treatment

**DOI:** 10.1097/MD.0000000000032899

**Published:** 2023-02-22

**Authors:** Yaser Alduais, Haijun Zhang, Fan Fan, Jing Chen, Baoan Chen

**Affiliations:** a Department of Hematology and Oncology, School of Medicine, Zhongda Hospital, Southeast University, Nanjing, Jiangsu, China; b Department of Biochemistry and Molecular Biology, School of Medicine & Holistic Integrative Medicine, Nanjing University of Chinese Medicine, Nanjing, Jiangsu, China.

**Keywords:** non-small cell lung cancer, treatment

## Abstract

Lung cancer remains the leading cause of cancer deaths. Non-small cell lung cancer (NSCLC) is the most frequent subtype of lung cancer. Surgery, radiation, chemotherapy, immunotherapy, or molecularly targeted therapy is used to treat NSCLC. Nevertheless, many patients who accept surgery likely develop distant metastases or local recurrence. In recent years, targeted treatments and immunotherapy have achieved improvement at a breakneck pace. Therapy must be customized for each patient based on the specific medical condition, as well as other variables. It is critical to have an accurate NSCLC sub-classification for tailored treatment, according to the latest World Health Organization standards.

## 1. Introduction

Lung cancer represents the leading cause of cancer-related deaths. The globally increasing tobacco use accounts for a rise in lung cancer mortality all over the world, notably in Asia, as predicted by the World Health Organization (WHO).

## 2. Factors

Several environmental and behavioral factors have been correlated with the development of lung cancer. Smoking leads to 85 to 90% of all lung cancer cases.^[1]^ Apart from tobacco smoking, second-hand smoke and family history as well as carcinogenic chemicals and heavy metals, such as radon gas, asbestos, arsenic, chromium, beryllium, and nickel, also highly increases the risk of lung cancer.^[2,3]^ In addition, pulmonary fibrosis, HIV infection, and alcohol use are also responsible for the carcinogenesis of lung cancer.^[4,5]^

## 3. Classification

Lung cancer is classified into 2 main types: non-small cell lung cancer (NSCLC, 85% of patients) and SCLC (15% of patients).

The 3 main types of NSCLC categorized by the WHO are: adenocarcinoma (40%), squamous cell carcinoma (25–30%), and large cell carcinoma (5–10%).^[6,7]^

There are also several combinations and variants of clinical subtypes.

## 4. Diagnosis

Often, NSCLC is not detected until this disease has developed into an advanced state.^[8,9]^ Cough is the most prevalent symptom, occurring in 50 to 75% of patients, followed by hemoptysis, dyspepsia, and achy chest.^[9]^

Positron emission tomography and computed tomography (CT) provide a more accurate classification of N-stage diagnosis.^[10,11]^ As validated by the examination of a secondary goal from another randomized research, each positive node on positron emission tomography-CT has to be sampled.^[12]^

CT and magnetic resonance imaging scans are frequently applied to image the brains of individuals who undergo curative treatments or exhibit signs/symptoms of brain metastases.^[13]^

It is also vital to collect adequate tissue material, according to the International Association for the Study of Lung Cancer, the American Thoracic Society, and the European Respiratory Society’s new lung cancer classification.^[14]^ The possibility of identifying mutations and tailored therapy has ramifications for all suspected lung tumors during the first examination.

Currently, positive predictive value (2.4–7.5%) is the best technique to predict hemoptysis, one of the major symptoms of lung cancer.^[15]^ The eighth edition of the staging system offers prognostic analysis of each tumor node metastasis descriptor to establish a more exact categorization.^[16]^

A liquid biopsy is also utilized to detect cancer biomarkers such as circulating tumor DNA, microRNA, and circulating tumor cells. In the Noninvasive versus Invasive Lung Evaluation trial of 282 patients with untreated NSCLC, circulating tumor DNA testing, as a less intrusive approach, is applied, increasing biomarker discovery rates by 48% compared to tissue analysis alone.^[17]^

## 5. Treatment

Surgery, radiation, chemotherapy, immunotherapy, or molecularly targeted therapy is used to treat NSCLC. Depending on the patient’s general health and disease stage, medically stable patients with stages I, II, and IIIA of NSCLC (usually when N2 lymph node disease is discovered during surgery) need to accept curative surgical excision. After 5 years, adjuvant platinum-based chemotherapy is suggested for patients in stages II to IIIA, in spite of great concerns about the high rates of recurrence and toxicity.^[18]^ Moreover, chemotherapy and immunotherapy are used to treat NSCLC patients at stage III.^[16]^ Molecularly targeted therapy is offered to improve survival in patients with NSCLC.^[19]^ In recent years, targeted treatments and immunotherapy have achieved significant improvements at a breakneck pace.

## 6. Surgery

Surgical resection is offered to remove the tumor from the lung and metastatic lymph nodes. The tumor must be removed from the border or margin of surrounding healthy lung tissues. When no malignancy is detected in the healthy tissue around the tumor, a “negative margin” is signified.

NSCLC is treated surgically in several ways, including lobectomy (the surgical removal of a lobe of the lungs), a wedge resection (the surgical removal of the tumor combined with the preservation of healthy lung tissue when the whole lung lobe is unable to be removed), segmentectomy (another approach when a full lung lobe cannot be removed and often applied for lung tissues and lymph nodes than a resection of a wedge), and pneumonectomy (the removal of the whole lung if the tumor is near the heart). The risk of a pneumonectomy is higher than that of a lobectomy.^[20–22]^

Nevertheless, many patients who undergo surgery are likely to develop distant metastases or local recurrence,^[23]^ so they need to take adjuvant therapy, such as radiation therapy, chemotherapy, and targeted therapy. After surgery, patients with Stage IIA, IIB, and IIIA NSCLC usually receive chemotherapy to kill any remaining cancer cells.^[24]^

## 7. Radiation therapy

High-energy beams used in radiotherapy could break DNA in cancer cells, consequently killing cancer cells. This treatment can inhibit tumor progression or eliminate tumors in specific parts of the human body. For NSCLC patients who are insensitive to surgery or chemotherapy, radiotherapy can be used as part of the palliative treatment to improve their quality of life.^[25]^

Patients who have possibly resectable tumors but are not eligible for surgery, or patients at inoperable Stage I who have sufficient pulmonary reserve, may be candidates for curative radiation therapy.^[26,27]^

Stereotactic body radiation therapy is used for early-stage NSCLC patients who only have a single small nodule in the lung without any metastases. According to some studies, stereotactic body radiation therapy has a lower cost, offers greater convenience for patients, and leads to better 2-year overall survival rates.^[28–30]^

## 8. Chemotherapy

Chemotherapy is a kind of medication that destroys cancer cells by inhibiting growth, division, and proliferation of tumor cells. It has been proven to prolong the life span and improve the life quality of patients with lung cancer at any stage.

The first-line treatment for Stage IV NSCLC is cytotoxic chemotherapy, which is influenced by histology, age, comorbidities, and performance status.^[31]^

Large-scale French research compared surgery combined with preoperative chemotherapy (mitomycin, ifosfamide, and cisplatin) to surgery alone, finding no benefit with neoadjuvant therapy. However, a subset analysis demonstrated that preoperative chemotherapy has a survival benefit for patients with N0 and N1 disease but not N2 disease.^[32]^

As reported, chemotherapy or epidermal growth factor receptor (EGFR) kinase inhibitors improve the median survival of patients with advanced cancer, but the overall survival remains poor.^[33,34]^

## 9. Targeted therapy

Tumor genes, proteins, or the tissue environment that promote tumor development and survival are the focus of targeted therapy. This treatment slows the development and spread of cancer cells, but will not adversely affect healthy cells. Targets for malignancies are not the same.^[35]^ In order to choose the most effective therapeutic target, patients will accept tests to identify the genes, proteins, and other factors in tumors. In certain types of lung cancer, aberrant proteins are discovered in abnormally high concentrations inside cancer cells. Additionally, research investigations continue to uncover novel information regarding particular biological targets and novel therapy methods.

### EGFR inhibitors.

In the US, around 10% to 15% of all lung cancer patients test positive for the EGFR mutation, and Asian patients have a higher EGFR mutation frequency (51.4% overall) in tumors.^[36,37]^ Researchers discovered that drugs that inhibit the EGFR pathway may be helpful to prevent or restrain the growth of lung cancer cells with EGFR mutation. A number of EGFR inhibitors have been approved by the United States Food and Drug Administration (FDA), including afatinib (GILOTRIF), entrectinib (ROZLYTREK), erlotinib (TAREVA), gefitinib (IRESSA), and osimmertinib (TAGRISSO).

New data showed that patients who were treated with osimertinib have higher progression-free survival (PFS) compared to those who accepted first-line treatment (gefitinib or erlotinib), so osimertinib was elevated to first-line therapy for EGFR-mutant NSCLC.^[38]^ The treatment of EGFR-mutant NSCLC has changed dramatically in the past few years.^[39]^

### Anaplastic lymphoma kinase (ALK) inhibitors and ROS1 fusion.

ALK plays a promoting role in cancer cell proliferation. ALK translocation is observed in around 5% of NSCLC patients.^[40]^ ROS1 fusions or ROS1 mutations are very rare and may impair cell development and differentiation. ROS1 fusions are observed in 1 to 2% of NSCLC patients.^[41]^

Drugs that target ALK and ROS1 mutations: alectinib (ALECENSA), brigatinib (ALUNBRIG), ceritinib (ZYKADIA), lorlatinib (LORBRENA), crizotinib (XALKORI), and entrectinib (ROZLYTREK).^[40,42–45]^

### Drugs targeting KRAS G12C mutations.

KRAS G12C is found to be the main genetic mutation in NSCLC patients. About 20 to 25% of lung cancer patients have a KRAS mutation. Sotorasib (LUMAKRAS) is the only approved drug for KRAS G12C mutations.

### Drugs targeting NTRK fusion.

NTRK fusion is a kind of genetic mutation that could be seen in a wide range of tumors. Such mutation could promote cancer cell growth. Lung cancer patients are far less likely to have NTRK fusion (<1 %).

Larotrectinib, an FDA-approved oral tropomyosin receptor tyrosine kinase inhibitor, is applicable to treat patients with advanced malignancies that have NTRK fusion without an acquired resistance mutation and who have no other treatment choices.^[46,47]^

### Drugs targeting BRAF V600E mutations.

Two percent of NSCLC patients have BRAF mutations, with the BRAF V600 mutation accounting for half of these cases. In a phase 2 study, vemurafenib, an oral small-molecule TKI, achieved a response rate of 42% and a median PFS of 7.3 months. The FDA has authorized dabrafenib and trametinib for patients with BRAF V600E mutations whose cancer has progressed during chemotherapy.^[48–50]^

### Drugs targeting MET exon 14 skipping.

The loss of MET exon 14 and enhanced MET signaling are caused by splicing site disruptions in the MET proto-oncogene. Tumor development, survival, invasion, and metastasis are all affected by these MET mutations, which are found in around 3 to 4% of NSCLC patients.^[51]^ Drugs approved to treat MET exon 14 skipping include capmatinib (TABRECTA) and tepotinib (TEPMETKO).^[52]^

### Drugs targeting RET fusion.

Up to 2% of all NSCLC patients have RET fusion. Pralsetinib (GAVRETO) and selpercatinib (RETEVMO) are 2 drugs authorized to treat NSCLC patients with RET fusion.^[53]^

### Drugs targeting vascular endothelial growth factor.

Angiogenesis therapy hampers the process of blood vessel formation. As tumors need nutrients carried by blood vessels to develop and spread, anti-angiogenesis medicines seek to “starve” the tumor of nutrition. The following anti-angiogenic agents may be used in the treatment of lung cancer: bevacizumab in combination with chemotherapy, and other agents targeting vascular endothelial growth factors such as ramucirumab combined with docetaxel.^[54,55]^

## 10. Immunotherapy

Immunotherapy or biological treatment is used to boost the inherent anti-cancer defense in human bodies. It utilizes natural or synthetic compounds to enhance, target, or restore immune system function. Recent advances in cancer treatment focus on developing drugs that target the immune system’s interaction with tumors. Immunotherapy could take effect regardless of mutation status in cancer cells and has fewer side effects. Clinical studies have indicated benefits from therapies targeting PD-1, CTLA4, and PD-L1.^[55]^

Immunotherapy for NSCLC patients might be a single immunotherapy drug, a combination of immunotherapy drugs, or immunotherapy combined with chemotherapy. Immunotherapy alone, or in combination with chemotherapy, is widely used to treat advanced NSCLC that is resistant to targeted treatment.

In the CheckMate 017 and 057 investigations, the PD-1 inhibitor nivolumab showed significant anticancer effectiveness in the extensively pretreated metastatic situation.^[56]^ An updated survival analysis found nivolumab continued to demonstrate a survival benefit versus docetaxel, exhibiting a 5-fold increase in overall survival rate, with no new safety signals,^[56–59]^ for which the FDA authorized nivolumab for treating patients with metastatic NSCLC (both squamous and nonsquamous histologies).

### Drugs that block the PD-1 pathway.

The PD-1 pathway may be critical to the immune system’s capacity to inhibit tumor progression. Anti-PD-1 and anti-PD-L1 antibodies have been shown to slow or halt the progression of NSCLC. Three immunotherapy medicines for NSCLC have been authorized by the FDA, including atezolizumab (TECENTRIQ), nivolumab (OPDIVO), and pembrolizumab (KEYTRUDA).

Nivolumab is regularly used as a second-line treatment for patients with metastatic NSCLC who get worse during or after platinum-based chemotherapy since it is linked with better survival and lower toxicity than docetaxel.^[57–59]^

Expedited approval of the immunotherapy drug pembrolizumab has been granted for NSCLC patients whose tumors express PD-L1 (>50% staining as verified by an FDA-approved test) at the advanced stage or with unresectable tumors.^[60]^

### Drugs that block the CTLA-4 pathway.

The CTLA-4 pathway is another immune signaling pathway that could be targeted. The FDA has authorized one medicine that inhibits NSCLC progression: ipilimumab (YERVOY). Ipilimumab in combination with PD-1 pathway inhibitor nivolumab may be used as part of a chemotherapy regimen.^[61]^ This therapy has a lower risk of major side effects than chemotherapy, despite the fact that different types of immunotherapy might cause different sorts of side effects. Side effects such as skin reactions and flu-like symptoms are possible with immunotherapy. Shortness of breath and weight fluctuations may also occur.^[62]^

## 11. Conclusions

Depending on different stages, varied treatment options are offered to NSCLC patients. Therapy must be customized for each patient based on specific medical condition and other variables.

Combining immunotherapy with targeted therapy brings innovation to the treatment of patients with NSCLC. Regardless of whether immunotherapy is administered alone or in conjunction with chemotherapy, it has become the standard first-line treatment.

Currently, the overall PFS rate for lung cancer patients remains poor, in spite of great breakthroughs in targeted therapy and immunotherapy. According to the latest WHO standards, it is critical to have an accurate NSCLC sub-classification for tailored treatment.

## Acknowledgments

Sincere thanks for all support.

## Author contributions

**Conceptualization:** Yaser Alduais.

**Methodology:** Haijun Zhang.

**Project administration:** Yaser Alduais.

**Resources:** Baoan Chen.

**Validation:** Jing Chen.

**Visualization:** Jing Chen.

**Writing – original draft:** Yaser Alduais, Fan Fan, Jing Chen, Baoan Chen.

**Writing – review & editing:** Yaser Alduais, Fan Fan, Jing Chen, Baoan Chen.
